# Cost and outcome of behavioural activation versus cognitive behaviour therapy for depression (COBRA): study protocol for a randomised controlled trial

**DOI:** 10.1186/1745-6215-15-29

**Published:** 2014-01-21

**Authors:** Shelley Rhodes, David A Richards, David Ekers, Dean McMillan, Sarah Byford, Paul A Farrand, Simon Gilbody, Steven D Hollon, Willem Kuyken, Christopher Martell, Heather A O’Mahen, Emer O’Neill, Nigel Reed, Rod S Taylor, Ed R Watkins, Kim A Wright

**Affiliations:** 1 University of Exeter Medical School, St Luke’s Campus, Exeter EX1 2 LU, UK; 2 Durham University/Tees Esk and Wear Valleys NHS Foundation Trust, Wolfson Research Institute for Health and Wellbeing, Durham University, Queen’s Campus, University Boulevard, Stockton on Tees, Durham TS17 6BH, UK; 3 Department of Health Sciences, Seebohm Rowntree Building, University of York, Heslington, York YO10 5DD, UK; 4 Institute of Psychiatry, Kings College London, De Crespigny Park, London SE5 8AF, UK; 5 School of Psychology, Sir Henry Wellcome Building for Mood Disorders Research, University of Exeter, Streatham Campus, Exeter EX4 4QG, UK; 6 Department of Psychology, 306 Wilson Hall, 21st Avenue South, Vanderbilt University, 2301 Vanderbilt Place, Nashville, TN 37240-7817, USA; 7 Department of Psychology, University of Wisconsin, Milwaukee, P.O. Box 413, Milwaukee WI 53201, USA; 8 Depression Alliance, 20 Great Dover Street, London SE1 4LX, UK; 9 Lived Experience Group, c/o School of Psychology, Sir Henry Wellcome Building for Mood Disorders Research, University of Exeter, Streatham Campus, Exeter EX4 4QG, UK; 10 University of Exeter Medical School, The Veysey Building, Salmon Pool Lane, Exeter EX2 4SG, UK

## Abstract

**Background:**

Cognitive behaviour therapy (CBT) is an effective treatment for depression. However, CBT is a complex therapy that requires highly trained and qualified practitioners, and its scalability is therefore limited by the costs of training and employing sufficient therapists to meet demand. Behavioural activation (BA) is a psychological treatment for depression that may be an effective alternative to CBT and, because it is simpler, might also be delivered by less highly trained and specialised mental health workers.

**Methods/Design:**

COBRA is a two-arm, non-inferiority, patient-level randomised controlled trial, including clinical, economic, and process evaluations comparing CBT delivered by highly trained professional therapists to BA delivered by junior professional or para-professional mental health workers to establish whether the clinical effectiveness of BA is non-inferior to CBT and if BA is cost effective compared to CBT. Four hundred and forty patients with major depressive disorder will be recruited through screening in primary care. We will analyse for non-inferiority in per-protocol and intention-to-treat populations. Our primary outcome will be severity of depression symptoms (Patient Health Questionnaire-9) at 12 months follow-up. Secondary outcomes will be clinically significant change and severity of depression at 18 months, and anxiety (General Anxiety Disorder-7 questionnaire) and health-related quality of life (Short-Form Health Survey-36) at 12 and 18 months. Our economic evaluation will take the United Kingdom National Health Service/Personal Social Services perspective to include costs of the interventions, health and social care services used, plus productivity losses. Cost-effectiveness will explored in terms of quality-adjusted life years using the EuroQol-5D measure of health-related quality of life.

**Discussion:**

The clinical and economic outcomes of this trial will provide the evidence to help policy makers, clinicians and guideline developers decide on the merits of including BA as a first-line treatment of depression.

**Trial registration:**

Current Controlled Trials ISRCTN27473954

## Background

Clinical depression is one of the most common and debilitating of the psychiatric disorders. It accounts for the greatest burden of disease among all mental health problems, and is expected to become the second largest amongst all general health problems by 2020 [1]. Lifetime prevalence has been estimated at 16.2% and rates of co-morbidity and risk for suicide are high [2-4]. Depression is recurrent, with over three-quarters of all people who recover from one episode going on to have at least one more [5]. Without treatment many cases become chronic, lasting over 2 years in one-third of individuals. In the UK, the annual costs of depression and anxiety to the economy are estimated at £17bn in lost output and direct health care costs, with a £9bn impact on the Exchequer through benefit payments and lost tax receipts [6].

Antidepressant medication (ADM) and cognitive behaviour therapy (CBT) are the two treatments with most evidence of effectiveness; both are recommended by the National Institute for Health and Care Excellence (NICE) [7]. Problems with ADM include side effects, poor patient adherence and relapse risk on ADM discontinuation. Service-user organisations and policy think tanks advocate greater availability of psychological therapies, which many people prefer [8]. CBT, which is of similar efficacy to ADM [9], has several advantages: 1) it reflects the desire of many service users for non-pharmacological treatment; 2) it has no physical side-effects; and 3) it modifies the illness trajectory in that benefits continue after the end of treatment thereby reducing rates of recurrence [10]. However, CBT has several limitations: 1) its complexity may make it difficult to learn to implement in a competent fashion; 2) there is some evidence that its efficacy is dependent upon the skill of the individual practitioner; 3) patients are required to learn high-level skills; and 4) the high costs of training and employing sufficient therapists limits access to CBT.

As a consequence of the problems above, many people do not receive adequate treatment and even, when treatment is given, many respond only partially or not at all [11]. For example, despite the recent UK government initiative in England - ‘Improving Access to Psychological Therapies’ (IAPT: http://www.iapt.nhs.uk/) - no more than 15% of people with depression will receive National Health Service (NHS)-delivered CBT and only 50% will recover [12]. It is therefore important to continue to test promising new treatments, especially if: there are indications that such treatments reduce the risk of symptom return; are applicable to a wide range of depressed people including those with high severity; are easy to implement in clinical practice and are therefore potentially more accessible [13]; and are a cost-effective use of resources. Indeed, in order to meet public and professional expectations, health services require simple, equivalently effective, easily implemented psychological treatments for depression that can be delivered by less specialist (albeit appropriately competent) junior or para-professional health workers to treat many more people with depression in a more cost-effective manner.

Behavioural activation (BA) is a psychological treatment based on behavioural theory that alleviates depression by focusing directly on changing behaviour [14-16]. This theory states that depression is maintained in part by avoidance of normal activities. As people withdraw and disrupt their basic routines, they become isolated from positive reinforcement opportunities in their environment. The combination of increased negative reinforcement with reduced positive reinforcement results in a cycle of depressed mood, decreased activity and avoidance which maintains depression [15]. BA systematically disrupts this cycle, initiating approach-oriented behaviours in the presence of negative mood, when people’s natural tendency is to withdraw or avoid [17,18]. Although CBT incorporates some behavioural elements, these commonly focus on increasing rewarding activity and initiating behavioural experiments to test specific beliefs. In contrast, BA targets avoidance from a contextual, functional approach not found in CBT - that is, BA focuses on understanding the function of behaviour and replacing it accordingly. BA also explicitly prioritises the treatment of negatively reinforced avoidance and rumination. Furthermore, because the BA rationale is simpler, it should be easier to understand and operationalise for both patients and mental health workers than CBT, where activity is also increased but the primary techniques focus on changing maladaptive beliefs [19]. Moreover, there is some evidence that CBT is less effective when delivered by less competent therapists [9,20].

In the UK, CBT is delivered by professionally qualified senior mental health workers (mainly clinical psychology, nursing, occupational therapy, social work or counselling), who have obtained a further 1 year full time post-graduate qualification in CBT. Their training is long and expensive and their employment grade is costly compared to junior professionals or para-professional mental health workers who deliver much of the routine mental health care in the UK. The relative simplicity of BA treatment may make it easier and cheaper to train para-professional mental health workers in its application than CBT, the argument of ‘parsimony’ first advanced by one of the early proponents of this approach, Neil Jacobson, more than 10 years ago [15].

### Limitations of previous trials

We conducted a meta-analysis of randomised controlled trials of BA [21] where we first found a clinical effect size in terms of a reduced depression score of −0.70 SD units from twelve studies (n = 459; 95% CI −1.00 to −0.39; *P* < 0.001) comparing behavioural treatments to controls using experienced therapists. We then found twelve studies comparing behavioural treatments with CBT (n = 476) and showed that behavioural treatments had equivalent outcomes to CBT (pooled SMD 0.08; 95% CI −0.14 to 0.30, *P* = 0.46). Meta regression of results exploring association between baseline severity and effect size identified a statistically significant result in favour of BA (meta-regression b-coefficient −0.05; 95% CI −0.10 to −0.01; *P* = 0.04).

However, many of the trials were of limited methodological quality, all were under-powered for comparing treatments, and most did not utilise diagnostic interviews for trial inclusion. Treatments in many cases did not conform to modern clinical protocols for BA. Long-term outcomes were rarely reported, with average follow-up only to 4 months. These results have been replicated in two recent Cochrane reviews of behavioural therapies [22,23] which concluded that there was only low- to moderate-quality evidence that behavioural therapies and other psychological therapies were equally effective and called for ‘Studies recruiting larger samples with improved reporting of design and fidelity to treatment’ to ‘improve the quality of the evidence’ [22] (page 2).

Therefore, the existing trial data are insufficient to provide certainty that BA should be a first-line treatment for depression and these limitations led to NICE in the UK regarding the evidence for BA as equivocal and of insufficient strength to recommend BA for first-line routine NHS depression treatment [7]. Consequently, NICE made a clear research recommendation “to establish whether behavioural activation is an effective alternative to CBT” using a study “large enough to determine the presence or absence of clinically important effects using a non-inferiority design” [7] (page 256).

### Pilot work preceding this trial

In order to test uncertainties around our main objectives, we piloted BA in a phase II randomised controlled trial to examine whether mental health workers without previous specialist training in psychological therapy can effectively treat depressed people using BA [24]. We compared BA against usual care. Relatively junior NHS mental health workers ('band 5' - equivalent to a basic grade qualified mental health nurse) with no previous formal training or experience in psychotherapy delivered BA. These workers received 5 days training in BA and subsequent 1-hour clinical supervision fortnightly from a clinical nurse consultant or trained psychotherapist. Intention-to-treat (ITT) analyses indicated a difference in favour of BA of −15.79 (n = 47; 95% CI −24.55 to −7.02) on depression (Beck Depression Inventory-II), an effect size of −1.15 SD units (95% CI −0.45 to −1.85). We also found a quality-adjusted life year (QALY) difference in favour of behavioural activation of 0.20 (95% CI 0.01 to 0.39, *P* = 0.042), incremental cost-effectiveness ratio of £5,756 per QALY and a 97% probability that behavioural activation is more cost-effective at a threshold value of £20,000 [25].

### Objectives

1 What is the clinical effectiveness of BA compared to CBT for depressed adults in terms of depression treatment response at 12 and 18 months?

2 What is the cost-effectiveness of BA compared to CBT at 18 months?

It is hypothesised that BA is non-inferior compared to CBT in reducing depression severity but that BA will be less costly and thus more cost-effective than CBT.

In addition, we will undertake a secondary process evaluation to investigate the moderating, mediating and procedural factors in BA and CBT that influence outcome.

### Methods/Design

COBRA is a two-arm, non-inferiority, patient-level randomised controlled trial, including clinical, economic and process evaluation for people with depression. We will test the effectiveness of a psychological intervention for depression - BA - against CBT, the current gold standard evidence-based psychological treatment for depression. The rationale for a non-inferiority trial is that we wish to establish whether the clinical effectiveness of BA is not substantially inferior to CBT and to determine if BA represents a viable first choice of treatment in the management of depression. Accordingly, we have powered our trial on the basis of clinical non-inferiority, and will analyse our data accordingly [26,27].

### Setting and participants

We will recruit participants from the electronic case records of primary care general practices in three UK sites: Devon, Durham and Leeds. Eligible participants will be aged 18 and older with DSM Major Depressive Disorder assessed by standard clinical interview (Structured Clinical Interview for Depression; SCID) [28]. People will be excluded if they are alcohol or drug dependent, are currently acutely suicidal or have made a suicide attempt in the previous 2 months, are cognitively impaired, or have a bipolar disorder or psychosis/psychotic symptoms. We will also exclude people currently in receipt of psychological therapy.

### Randomisation, concealment of allocation, and blinding

Participants will be allocated in a 1:1 ratio to either the BA or CBT arms stratified according to their symptom severity on the Patient Health Questionnaire 9 (PHQ-9) [29] (PHQ-9 <19 versus ≥19), antidepressant use (currently using anti-depressants or not) and recruitment site. A computer-based system will allocate the first 20 participants to each arm on a truly random basis. For subsequent participants, allocation will be minimised to maximise the likelihood of balance in stratification variables across the two study arms. Concealment will be ensured by use of an externally administered password-protected trial website and retaining a stochastic element to the minimisation algorithm. The computer-based allocation and website will be setup and maintained by the accredited Peninsula Clinical Trials Unit, independent of the trial. The participant’s details will be sent to the relevant mental health worker to alert them to contact this person and begin treatment.

All research measures will be applied to both groups of participants equally. Researchers will be blind to group allocation, which will occur after baseline assessments. At follow-up, researchers will be instructed to maintain blindness by reminding participants of the confidential nature of their treatment and the need not to discuss this with researchers. We will test blindness by asking researchers to indicate at follow-up which treatment they believe the participants received and analyse any correlation with outcome.

### Recruitment

**Figure 1:**
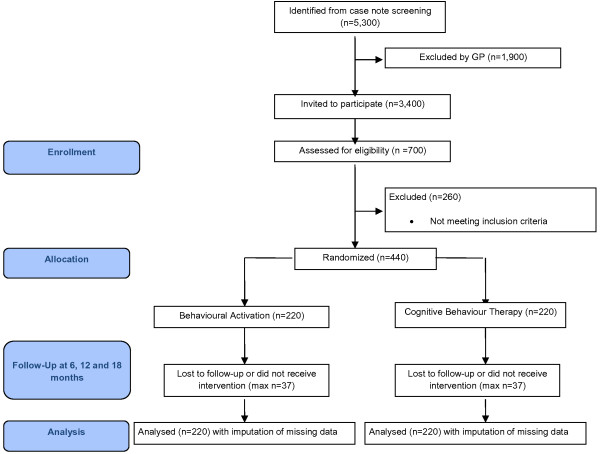
**Consort diagram describing flow of patients through study.** GP, general practitioner.

Randomised controlled trials are vulnerable to selection bias and threats to external validity if there are systematic differences in behaviour between referring clinicians. We will minimise this potential bias by recruiting participants through searching general practice records and referral logs from primary care to local depression and anxiety treatment services, rather than by direct referral from general practitioners (GPs). We will identify suitable participants by examining electronic case records for all patients in each general practice or treatment service. The GP search will be limited to people seen by their GPs in the previous 2 months who have been allocated at least one identification code in their electronic records. Several such codes are widely used by general practitioners to classify patients. Practice staff or Research Network Clinical Studies Officers will conduct searches. The list of potentially suitable participants will be reviewed by GPs to identify any patients whom have known exclusion criteria. The remaining patients will be written to by their GP practice, inviting them to take part in the study. For patients already referred to local psychological therapies services, we will contact those on the waiting list. A short participant information sheet, stamped addressed envelope and a ‘Permission for Researcher to Contact’ form to allow a researcher to contact them, will accompany letters. If potential participants do not return the form, they will be contacted by telephone by practice staff, practice based Research Network Clinical Studies Officers or service administrators to check they have received the letter and asking them if they wish to participate in the COBRA trial. Potential participants identified by either written or telephone routes will be initially telephone screened by researchers to confirm the presence of depressive symptoms, and to explain the trial fully. If positive on the screen, potentially eligible participants will be interviewed face-to-face by researchers to confirm eligibility, take consent, conduct a diagnostic interview and collect baseline measures. Eligible, fully informed and consenting participants will then be entered into the study and randomisation (See Figure 1).

### Trial interventions

We have specified our BA and CBT for depression intervention protocols in line with (a) the original treatment protocols [9,15,17-19,30,31] and (b) NICE recommendations [7] for duration and frequency of BA and CBT. To recognise realities of real world clinical presentations, our protocols will include behavioural and cognitive strategies for managing comorbidity, particularly anxiety, where this is present in addition to depression. Participants will receive a maximum of 20 sessions over 16 weeks with the option of four additional booster sessions [7].

### Behavioural activation

The overall goal of BA is to re-engage participants with stable and diverse sources of positive reinforcement from their environment and to develop depression management strategies for future use. Mental health workers delivering BA will follow a revised treatment manual based on that used in our Phase II trial [24] and previous international studies [30], incorporating recommendations from NICE Guidelines [7] and advice from our international collaborators, Martell and Dimidjan. Sessions will be face-to-face, of 1-hour duration, with the option of being conducted up to twice weekly over the first 2 months and weekly thereafter. They will consist of a structured programme increasing contact with potentially antidepressant environmental reinforcers through scheduling and reducing the frequency of negatively reinforced avoidant behaviours. The central behavioural technique will be functional analysis of the participant’s problems, based on a shared formulation drawn from the behavioural model in the early stages of treatment, thereafter developed with the patient throughout their sessions. Specific BA techniques include the use of a functional analytical approach to develop a shared understanding with patients of behaviours that interfere with meaningful, goal-oriented behaviours and include self-monitoring, identifying ‘depressed behaviours’, developing alternative goal-orientated behaviours and scheduling. In addition, the role of avoidance and rumination will be addressed through functional analysis and alternative response development incorporating recent trial evidence [32].

Workers will be selected from NHS band 5 mental health workers such as mental health nurses and para-professional Psychological Wellbeing Practitioners [33], and will receive 5 days training in BA. In line with the programme developed and tested in our Phase II trial [24], training will focus upon the rationale and skills required to deliver the BA protocol for depression and include sections on behavioural learning theory and its application to depression, developing individualised BA formulations and specific techniques used in sessions. Training will be a mix of presentation and role-play with repeated practise and feedback. Workers will be competency-assessed at the end of training using standardised marking criteria consistent with the BA protocol and further training given if competency is not demonstrated in practical clinical exercises. BA workers will receive subsequent 1-hour clinical supervision fortnightly from the three site leads or other members of the trial team, clinically qualified in BA.

### Cognitive behaviour therapy

The overall goal of CBT is to alter the symptomatic expression of depression and reduce risk for subsequent episodes by correcting the negative beliefs, maladaptive information processing and behavioural patterns presumed to underlie the depression. Therapists delivering CBT will follow a treatment protocol based on the standard manuals published by Beck and colleagues [19,31], the manual used in our recent trial [34] and additional advice and training resources from our US collaborator, Steven Hollon. Sessions will be face-to-face, of 1-hour duration, with the option of being conducted up to twice weekly over the first 2 months and weekly thereafter. They will consist of a structured, collaborative programme. Treatment begins with agreeing a problem list and goals for therapy, patients learning the CBT model, behavioural change techniques, and moves on to identifying and modifying negative automatic thoughts, maladaptive beliefs and, if indicated, underlying core beliefs. In later sessions, learning is translated to anticipating and practicing the management of stressors that could provoke relapse in the future. Specific CBT techniques include scheduling activity and mastery behaviours, the use of thought records and modifying maladaptive beliefs and rumination content. The behavioural elements in CBT focus on increasing activity with practical behavioural experiments to test specific cognitive beliefs. CBT will not take the contextual, functional analytical approach of the BA trial arm.

CBT will be delivered by senior mental health workers with a specialist postgraduate diploma in ‘high-intensity’ CBT from an accredited University course. These workers are employed at NHS band 7. They will also receive a 5-day orientation training to the specific CBT protocol [19,31], including its adaptation for co-morbidities, cognitive theory of depression, developing individualised cognitive formulations and specific techniques used in sessions. Therapists will be competency assessed at the end of training using standardised marking criteria consistent with the CBT protocol and further training given if competency is not demonstrated. CBT therapists will receive subsequent 1-hour clinical supervision fortnightly from established supervisors in the three sites with advice from other members of the trial team, clinically qualified in CBT.

### Intervention fidelity

We will assess the quality of and adherence to BA and CBT clinical protocols using audiotapes of therapy sessions. A random sample of tapes, stratified by therapist, therapy session and intervention will be sent to independent experts in both treatments for competence rating using the Cognitive Therapy Scale - Revised [35] for CBT and the Quality of Behavioral Activation Scale [36] for BA.

### Outcomes

We will conduct follow-up assessments at 6, 12, and 18 months post-baseline assessment. Our primary outcome will be self-reported depression severity and symptomatology as measured by the PHQ-9 [29] at 12 months. The PHQ-9 is a nine-item questionnaire that records the core symptoms of depression with established excellent specificity and sensitivity characteristics in a UK population [37]. Our secondary outcomes will be DSM Major Depressive Disorder status and number of depression free days between follow-ups, assessed by standard clinical interview (SCID) [28], anxiety assessed by the Generalised Anxiety Disorder-7 [38], and health-related quality of life (Short-Form Health Survey-36) [39].

### Economic data

We will calculate QALYs using the EuroQol-5D (EQ-5D)-3 L measure of health-related quality of life [40]. The EQ-5D consists of five dimensions in the domains of mobility, self-care, usual activities, pain/discomfort and anxiety/depression, each scored on three levels (no problems, some problems or extreme problems) and classifies individuals into one of 243 health states. Health states are converted into a single summary index by applying weights to each level in each dimension derived from the valuation of EQ-5D health states in adult general population samples [41]. We will collect participants’ use of BA and CBT from clinical records, with information on additional resources involved (for example, training, preparation, supervision and so forth) collected directly from therapists. We will measure all other health and social care services used, including medication prescription and use, and productivity losses using the Adult Service Use Schedule, designed on the basis of previous evidence of service use in depressed populations [42]. We will also measure productivity losses using the absenteeism and presenteeism questions of the World Health Organization’s Heath and Work Performance Questionnaire [43]. We will calculate intervention costs using a standard micro-costing (bottom-up) approach [44], which will be based on therapist salaries plus employers costs (national insurance and superannuation contributions) plus appropriate capital, administrative and managerial overheads. We will take costs for NHS hospital contacts from NHS reference costs and apply nationally applicable unit costs to all community health and social care contacts [45]. We will take the cost of medications from the British National Formulary [46].

### Process data

In addition to information on age of depression onset and number of previous episodes collected using the SCID [28], we will also collect data on changes in specific behaviour (Behavioural Activation for Depression Scale) [47], changes in beliefs (Dysfunctional Attitude Scale) [48], ruminative response style (Ruminative Response Scale of the Response Styles Questionnaire) [49], hedonic tone (Snaith-Hamilton Pleasure Scale) [50], acceptability of BA and CBT for participants and clinicians (assessed with qualitative process studies), and per-protocol (PP) treatment adherence (from therapist case records). We will collect qualitative data via semi-structured interviews and written responses to access participants’ accounts of the mechanisms and impacts of treatment. At the end of treatment, participants will write short accounts of their experiences of, and perceived impacts of, treatment in response to open-ended questions. Additionally, we will undertake semi-structured interviews designed to obtain a more in-depth understanding of the on-going mechanisms and impact of treatment with purposively sampled participants and therapists building on the analysis of the written accounts. Interviews will focus on the participants’ views of the role of cognitive and behavioural change strategies and broader impacts of treatment in participants’ lives. Integration with the quantitative process data will enhance understanding of change mechanisms that can improve these treatments’ potential efficacy [51,52].

### Sample size calculation

We have estimated the non-inferiority margin for the primary outcome (PHQ 9) using two potential approaches with reference to: (1) the effect size of historical trials comparing BA versus control; and (2) the published minimum clinically important difference for the primary outcome (PHQ-9) of 2.59 to 5.00 [53]. Based on our meta-analysis, BA was superior to control in depression score by a mean of 0.7 SD units (95% CI: 0.39 to 1.00) or 3.8 (2.1 to 5.4) on PHQ-9 score units (assuming an SD of 5.4 from Lowe and colleagues [53]). It has been proposed that non-inferiority margins be taken as ~0.5 × mean control effect size (that is, 0.5 × 3.8 = 1.90) or as the lower 95% limit of the control effect size (that is, 2.1) [54,55]. To ensure the adequacy of this trial to test non-inferiority between BA and CBT, we therefore examined a number of potential scenarios taking in account the potential uncertainty in the non-inferiority margin for the primary outcome (Table 1).

**Table 1:** Sample size calculation

**Approach**	**MICD**	**Power**	**Attrition rate**	Sample size per group*
50% BA-control effect size	1.90	90%	20%	220
50% BA-control effect size	1.90	80%	20%	160
LCI BA-control effect size	2.10	90%	20%	180
LCI BA-control effect size	2.10	80%	20%	135
Lower MCID	2.59	90%	20%	120
Lower MCID	2.59	80%	20%	90

*Calculated assuming Patient Health Questionnaire 9 SD of 5.4 [33] and 1-sided 2.5% alpha using NQuery v7.0 MTE0-6, NQuery Statistical Solutions, 4500 Airport Business Park, Cork, Ireland. BA, behavioural activation; LCI, lowest confidence interval; MCID, minimum clinically important difference.

We have selected a conservative non-inferiority margin of 1.90 and power of 90%. As a consequence, we will need to recruit a total of 440 participants to detect a between-group non-inferiority margin of 1.90 in PHQ-9 at 1-sided 2.5% alpha, allowing for 20% attrition caused by drop outs and protocol violators. Furthermore, although previous trials of CBT have shown little or no effects of clustering in outcome by therapists, even when delivering group CBT [56,57], if we were to assume a small therapist clustering effect (that is, intra-cluster correlation coefficient of 0.01) this sample size would still have 80% power for a non-inferiority margin of 1.90 in PHQ-9 at 1-sided 2.5% alpha, allowing for 20% attrition.

Our sample size is inflated by 20% for participant drop out to take account of those that exit the trial and refuse follow-up assessment, although our experience running large primary care trials of depression treatment is that attrition rates will be less than this. Therefore, we intend to recruit 440 participants to the trial, 220 per arm.

### Data analysis

All analyses will be carried out using an *a priori* statistical analysis plan prepared in the first 6 months of the trial and agreed with the Trial Management Group, Trial Steering Committee (TSC) and the Data Monitoring Committee.

Equivalence of baseline characteristics and outcomes in the two groups will be assessed descriptively. Since differences between randomised groups at baseline could have occurred by chance, no formal significance testing will be conducted. We will also undertake a descriptive analysis of the baseline patient characteristics according to the recruitment method (IAPT versus case note review).

We will analyse and report primary and secondary outcomes in accordance with reporting guidelines for non-inferiority and equivalence trials [27]. In a superiority trial, ITT analysis is conventionally used as the most conservative approach to minimise the possibility of a type I error - that is, falsely concluding that one treatment is superior to another. ITT includes data in the primary analysis from participants who drop out or violate the protocol to ensure differences between treatments under test are not falsely inflated and ensuring the most rigorous conditions apply before rejection of the null hypothesis (that is, treatment A is not superior to treatment B). However, in non-equivalence trials the null hypothesis is the opposite, and states that the experimental treatment is inferior to the reference treatment. CONSORT guidelines for such trials [27] recommend analyses to maximise the chances of finding a difference between treatments ensuring stringent conditions apply before rejection of the specific non-inferiority null hypothesis. Paradoxically, because conventional ITT analysis tends to bias towards not finding a difference, adopting an ITT approach could make the null-hypothesis easier to reject in non-inferiority trials by a “blurring of the difference between the treatment groups [which] increases the chance of finding equivalence” [58] - that is, a false non-inferiority result (type I error). Whilst the CONSORT guidelines recommend a PP approach (that is, analysis according to actual treatment received) as the conservative non-inferiority analysis option, given the potential biases of both PP and ITT analyses, we agree with the European Agency for the Evaluation of Medicinal Products that security of inference depends on both PP and ITT analyses demonstrating non-inferiority of the primary outcome [55]. We will, therefore, check for non-inferiority in PP and ITT populations, conducting sensitivity analyses on the primary outcome for PP and imputed ITT populations to check the security of inference of non-inferiority. We will also conduct sensitivity analyses using different definitions of PP adherence. We will include varying proportions of PP participants in these sensitivity analyses populations, depending on how much of each therapy they have received, ranging from 40 to 100% of planned therapy sessions. If non-inferiority is consistently shown by these analyses, we will proceed to assess superiority of CBT versus BA - that is, the CI lower bound lies above 0. If conclusions are inconsistent across analyses we will revert back to primacy of the PP analysis to confirm or refute the non-inferiority hypothesis.

The primary analysis will compare primary and secondary outcomes between BA and CBT groups at 12 months after randomisation adjusting for baseline outcome values and stratification variables (symptom severity, site, antidepressant use) and fitting therapist as a random effects variable. The one-sided 97.5% CI for the between-group difference will be estimated and non-inferiority of BA compared to CBT will be accepted (in a 0.025 level test) if the lower bound of the 97.5% CI lies within the non-inferiority margin of −1.90 in PHQ-9 score. If non-inferiority is shown, we will then test for superiority of CBT over BA (that is, lower bound of the 97.5% CI lies above 0). We will check for non-equivalence at all follow-up points using the same approach. Secondary analyses will be undertaken to compare groups at follow-up across 6, 12, and 18 months using a repeated-measures approach. The analysis will be extended to fit interaction terms to explore possible differences in treatment effect in baseline symptom severity and antidepressant usage. Sensitivity analysis, making different assumptions about the imputation model used, will be conducted for both primary and secondary analyses to assess the likely impact of missing data. Models will be fitted using generalized linear mixed models and undertaken in STATA v.11, STATA, StataCorp LP, 4905 Lakeway Drive, College Station, Texas 77845–4512, USA. We will also analyse the relative proportions of participants meeting criteria for “recovery” (proportions of participants with PHQ-9 scores ≤9) and “response” (50% reduction in scores from baseline) on the PHQ-9.

### Economic analysis

We will compare the costs and cost-effectiveness of BA and CBT at the final 18-month follow-up to capture the economic impact of events such as relapse, although we will conduct an initial preliminary analysis at 12 months to support the primary clinical analyses. Our primary analysis will take the NHS/personal social services perspective preferred by NICE [59]. The impact of productivity losses as a result of time off work, known to be a substantial cost in depression [60], will be explored in sensitivity analysis. In addition, narrower cost perspectives will be tested (for example, an intervention perspective and a mental health care perspective), to ensure that the NHS/personal social services perspective has not captured irrelevant costs that may hide the true impact of BA and CBT on service use.

Costs will be disaggregated to ensure the costs of each intervention and the costs to the different sectors (health, social care, employment) are clear. Although the distribution of costs is commonly skewed in populations of this kind, our analyses will compare mean costs using standard parametric t-tests with covariates for pre-specified baseline stratification factors plus baseline costs. We will confirm the robustness of the parametric tests using bias-corrected, non-parametric bootstrapping [61,62]. Whilst studies designed to test equivalence of effects are considered to be a legitimate situation in which a cost-minimisation analysis (where costs alone are compared given equal outcomes) may be appropriate [62], the same may not be true for non-inferiority designs. Even in situations where equivalence or non-inferiority are demonstrated, exploration of the joint distribution of costs and effects in a cost-effectiveness analysis is recommended to represent uncertainty [62] and to help interpret the economic results [58]. For these reasons, we will undertake a cost-effectiveness analysis irrespective of whether or not non-inferiority in the primary clinical outcome is demonstrated.

We will assess cost-effectiveness in terms of QALYs using the net benefit approach [63] with reference to Bosmans’ methods [58] for economic evaluations alongside equivalence or non-inferiority trials. Uncertainty around the cost and effectiveness estimates will be represented by cost-effectiveness acceptability curves [64,65]. We will generate a joint distribution of incremental mean costs and effects for the two therapies using non-parametric bootstrapping to explore the probability that each of the treatments is the optimal choice, subject to a range of possible maximum values (ceiling ratio) that a decision-maker might be willing to pay for an additional QALY gained. We will present cost-effectiveness acceptability curves by plotting these probabilities for a range of possible values of the ceiling ratio [66], a recommended decision-making approach to dealing with the uncertainty that exists around the estimates of expected costs and expected effects associated with the interventions under investigation and uncertainty regarding the maximum cost-effectiveness ratio that a decision-maker would consider acceptable [66,67].

### Process data analysis

Based on recent reviews [68], exploratory analyses will examine baseline variables that might moderate outcome at multiple time points (6, 12 and 18 months) across the two treatments (including depression severity, age of depression onset, number of previous episodes, and baseline levels of cognitive and behavioural dysfunction) using the approach set out by Kraemer and colleagues [51]. Although the power to detect moderate subgroup interactions will be low, we are primarily interested in exploring the possibility of large interactions that could inform subsequent clinical decision-making regarding treatment allocation.

Mediational analyses will investigate the hypothesised mechanisms of change (for BA: changes in specific behaviour such as reduced avoidance and rumination, learned capacity to apply behavioural principles to modify the environment; for CBT: changes in beliefs and underlying information processing style) pre-treatment to mid-treatment, and mid-treatment to post-treatment across the trial arms using approaches to testing mediation that allow multiple mediators in one model [51]. We will also analyse audio recordings of BA and CBT sessions to assess changes in putative mediators amongst patient and therapist within-session behaviour [52]. The effects of the mediators on outcome at 12 and 18 months will be modelled. This approach to examining mediation ensures that changes in putative mediators temporally precede changes in the primary outcome and allow baseline-to-post-treatment change in symptoms to be statistically controlled, necessary to rule out reverse causality. Analyses will include multivariate growth models including autoregressive and lagged terms, as well as recent developments in mediator analysis that use instrumental variables to account for the effect of unobserved confounding on mediators - we will follow precedent in using treatment allocation and its interaction with baseline measures as instrumental variables [69].

### Qualitative data analysis

Qualitative data will be analysed using a framework analysis [70] combining inductive and deductive approaches. Thematic frameworks will be developed from a combination of interview topics and data collected from participants to identify key concepts and themes. Interview transcripts will be examined thematically across the whole dataset as well as in the context of each interview, using a constant comparative analysis approach [71]. Data will be indexed, rearranged and mapped onto the identified themes and subthemes and interpreted and reanalyzed within the thematic framework to distil, interpret and structure component statements, the original transcripts being frequently revisited to clarify contextual meaning.

### Ethical issues

We will conduct the trial is such a way as to protect the human rights and dignity of the participants as reflected in the Helsinki Declaration [72]. Participants will not receive any financial inducement to participate. The study has received Multi-Centre Research Ethics Committee approval from the South West Research Ethics Committee in the UK. Local Research Ethics Committee and NHS Research and Development approvals have also been given for each recruitment site. To conform to data protection and freedom of information acts, all data will be stored securely and anonymised wherever possible. No published material will contain patient identifiable information.

### Obtaining informed consent from participants

We will determine informed consent by a two-phase consent process. Participants will receive a study information sheet in the post and a form seeking their permission to be contacted by a member of the research team, not at this stage to give consent to trial participation. The information leaflets will be produced using the current guidelines for researchers on writing information sheets and consent forms, posted on the UK ethics website (http://www.nres.nhs.uk/applications/guidance/consent-guidance-and-forms/) and informed by our consumer/lived experience user representatives. Participants who wish to partake in the trial will return their initial consent to be contacted form to the site research team. Full informed consent will only be obtained through an interview by a researcher where the information sheet is fully explained and where the opportunity to ask questions is given. The opportunity to withdraw from the trial will be fully explained. Researchers seeking consent will be fully trained and supervised by the chief investigator and site leads. Communication and recording systems will be set up to enable the trial team to monitor and act on participants’ wishes to withdraw from the trial.

### Anticipated risks and benefits

All participants will receive usual GP care, and therefore no treatment will be withheld to participants in this trial. Both arms are active psychological treatments with previously demonstrated efficacy and no known iatrogenic effects. This trial may in fact benefit individual participants, since CBT is not generally available for the majority of people with depression. By participating in this trial, participants will also receive an intensive level of monitoring such that any participants worsening or at suicidal risk will be identified and directed to appropriate care.

### Informing participants of anticipated risks and benefits

Participant information leaflets will provide potential participants with information about the possible benefits and known risks of taking part in the trial. Participants will be given the opportunity to discuss this issue with their GP or the trial manager prior to consenting. The trial manager will inform the participant if new information comes to light that may affect the participant’s willingness to participate in the trial.

### Suicide and suicide attempts

Inherent in the nature of the population under scrutiny is the risk of suicide. We will follow good clinical practice in monitoring for suicide risk during all research and clinical encounters with trial participants developed for our previous trials, for example [34,73]. Where any risk to participants due to expressed thoughts of suicide is encountered, we will report these directly to the GP (with the participant’s expressed permission), or if an acute risk is present we will seek advice from the GP immediately and follow locally established suicide risk management plans. Systems will be put into place to ensure that the chief investigator, trial manager and researchers will be informed should there be any risks to the participants’ safety.

### Patient and public involvement

The COBRA team work closely with UK mental health consumer organisations including RETHINK and Depression Alliance. The chief executive of Depression Alliance (O’Neill) is a co-investigator on this trial and has advised the team throughout. All sites have excellent local patient and public involvement (PPI) mechanisms led from the Exeter Mood Disorders Centre via our ‘Lived Experience Group’ - 20 people with personal experience of depression and its treatment. O’Neill and at least one member of the Lived Experience Group will attend all Trial Management Group meetings. Selection and writing of participant materials (trial information leaflets and consent forms; clinical materials; training materials) will be edited by this group. We will follow national good practice guidance for researchers on public involvement in research and the paying of PPI representatives actively involved in research at http://www.invo.org.uk. We will also work with our PPI representatives to ensure that our dissemination strategies are inclusive and accessible to other people who use services.

### Trial Steering Committee and Data Monitoring and Ethics Committee

A TSC will be set up and include an independent chair, an academic GP and at least two other independent members, along with the lead investigator and some other study collaborators. The TSC will meet at least once a year. A Data Monitoring and Ethics Committee (DMEC) committee will be set up and comprise an independent mental health statistician and clinician. The role of the DMEC is to review serious adverse events thought to be treatment related and look at outcome data regularly during data collection.

### Forecast execution dates

The preparatory period started in March 2012. Recruitment is running from September 2012 to June 2014. Follow up will last 18 months, at 6 (T1), 12 months (T2) and 18 (T3) months after randomisation. Data analysis and reporting will take another 6 months. The entire study period will last for 48 months.

## Discussion

This trial is designed to test the clinical and economic benefits of BA compared to the current gold standard psychological treatment of depression, CBT. Our non-inferiority design and analytical strategy will determine if BA is substantially inferior to this standard.

## Trial status

Recruitment commenced in September 2012 and is ongoing.

## Abbreviations

ADM: antidepressant medication; BA: behavioural activation; CBT: cognitive behaviour therapy; DMEC: Data Monitoring and Ethics Committee; EQ-5D: EuroQol-5D; GP: general practitioner; IAPT: Improving Access to Psychological Therapies; ITT: intention-to-treat; NHS: National Health Service; NICE: National Institute for Health and Care Excellence; PHQ-9: Patient Health Questionnaire 9; PP: per-protocol; PPI: patient and public involvement; QALY: quality-adjusted life year; SCID: Structured Clinical Interview for Depression; TSC: Trial Steering Committee.

## Competing interests

The authors declare that they have no competing interests.

## Authors’ contributions

DAR as chief investigator co-conceived the study, drafted the study protocol and study materials, and obtained ethics and NHS Research and Development approvals. DE, DMc, SB, PAF, SG, WK, HAO’M, EO’N, RST, ERW and KAW co-conceived and designed the study. DAR, SDH, CM, DE, DMc, PAF, WK, HAO’M, ERW and KAW designed the clinical interventions. EO’N and NR provide advice to the team on patient and public involvement. SR is involved in the study design and project management and helped to draft the manuscript with DAR. All other authors contributed to editing of the final manuscript. All authors read and approved the final manuscript.
